# An Analysis of the Inclusion of Mathematical Discourse Components in Arabic Mathematical Textbooks: The Case of Saudi Arabia

**DOI:** 10.3389/fpsyg.2020.534803

**Published:** 2020-11-20

**Authors:** Abdulwali H. Aldahmash, Naem M. Alamri

**Affiliations:** ^1^Excellent Research Center for Science and Mathematics Education, King Saud University, Riyadh, Saudi Arabia; ^2^College of Education, King Saud University, Riyadh, Saudi Arabia

**Keywords:** argumentative discourse, content analysis, discourse, mathematics textbooks, textbooks analysis

## Abstract

This study analyzes the content of 12th-grade mathematics textbooks and workbooks, based on their inclusion of mathematical discourse components. The mathematics textbooks and workbooks were used in a Saudi Arabian school, where students are transitioning from secondary education to university. The results revealed that Saudi Arabian school textbooks and workbooks did not appropriately include discourse components or discourse skills to help facilitate mathematical learning among students. Furthermore, these textbooks did not exceed level two of the four levels of inclusion. As a result, the inclusion was insufficient in helping students meaningfully understand mathematical concepts, become active students, and develop successful community leadership. This implies that mathematics textbooks and workbooks should be revised to include mathematical discourse so that this inclusion is more student directed than teacher directed.

## Introduction

Mathematics is a vital discipline in facilitating the mastery of science and technology. However, research has indicated that mathematics is a difficult field of study (Jablonka and Johansson, 2010; Simmers, 2011; Seifi et al., 2012). Therefore, it is difficult for students to understand mathematical concepts. This is due to the fact that mathematics is often not presented to students in a more appealing form, and this causes them to feel bored during mathematics lessons. With an unappealing mode of presentation, students are subsequently hindered in their ability to interact with the teacher or with each other.

The National Council of Teachers of Mathematics [NCTM] (2015) stressed that “discourse is the mathematical communication that occurs in a classroom and is considered as a tool for students to articulate their own ideas and seriously consider their peers’ mathematical perspectives as a way to construct mathematical understandings” (p. 1). In addition, Gee (1996) described mathematical discourse clearly, by saying: “A discourse is a socially accepted association among ways of using language, other symbolic expressions, and “artifacts,” of thinking, feeling, believing, valuing, and acting that can be used to identify oneself as a member of a socially meaningful group or “social network,” or to signal (that one is playing) a socially meaningful role” (p. 131). Gee (2005) also stressed that discourse goes beyond speech or writing as it is “not only a way of talking, acting, interacting, thinking, believing, reading, writing but also mathematical values, beliefs, and points of view” (p. 20). Furthermore, National Council of Teachers of Mathematics [NCTM] (1991) stated that discourse is “ways of representing, thinking, talking, agreeing, and disagreeing; the way ideas are exchanged and what the ideas entail; and as being shaped by the tasks in which students engage as well as by the nature of the learning environment” (p. 1). In addition, National Council of Teachers of Mathematics [NCTM] (2014) inferred that discourse deepens the students’ meaningful learning of mathematics, as well as improves the environment in which mathematics discourse occurs. Boston et al. (2017) stressed that teachers should know how to use discourse to build students mathematical thinking. In addition, students should be encouraged to have a dialogue with each other or talk to the class or discourse community, as this may enhance their learning of mathematics.

Previous research on mathematical discourse gained its importance from its ability of improving students’ conceptual understanding of mathematics concepts and mathematical reasoning (Novita et al., 2012; Surya et al., 2016). Other studies (Drake and Sherin, 2006; Rigelman, 2009) found that the mathematics curriculum used by teachers influences teacher decision in planning discourse in the classroom and that mathematical discourses were found to be appropriate in strengthening students’ mathematical ways of talking (Forman, 1996) and then ease obstacles that hinder mathematics learning among students (Gee, 1996).

Mathematical discourse inclusion in mathematics textbooks is worth studying, because it is highly important in support of mathematics learning, as well as the advancement of science and technology.

(1) It is always used in all facets of life; (2) all fields of study require appropriate math components; (3) it is a powerful means of communication, clear, and concise; (4) can be used to present information in a variety of ways; (5) it improves the ability to think logically, accuracy, and spatial awareness; and (6) it gives satisfaction to the efforts to solve challenging problems (Al-Najjar, 2009, p. 253).

Therefore, discourse should be given a major role in the objectives of teaching and learning mathematics in order to ease the difficulty of learning and teaching mathematics. As such, discourse should have been stressed as an important skill in school for students to develop (Lewison et al., 2006; Skolverket [National Agency of Education], 2011). In addition, discourse should be included in resources such as mathematics textbooks, classroom teaching, and in mathematics teachers’ professional development programs (Franke et al., 2007; Larsson and Ryve, 2011, 2012). This is important because textbooks, especially in Saudi Arabia, are the major resources, for both the teachers and the students (Stein et al., 2007; Jablonka and Johansson, 2010).

### The Role of Textbooks in Education

Multiple studies (Vincent and Stacey, 2008; Li et al., 2009; Van Stiphout, 2011) have stressed the importance of textbooks in mathematics education. They indicated that textbooks play a significant role in both teaching and learning (Valverde et al., 2002) because they are considered an energetic element of successful learning. Textbooks are defined simply as written books that are specified for teaching and learning. Venezky (1992) stressed that textbooks are constructed by using curricular guidelines that are specified to each curriculum type. Students in most countries, including Saudi Arabia, depend completely on textbooks to assist them with learning all curricula in school. Research revealed that textbooks are considered as a major resource for students as well as teachers. However, there is not enough research-based evidence regarding the framework through which students or teachers use mathematics textbooks in particular. Mikk (2000) noted that mathematics teachers highly rely on textbooks. In this regard, Valverde et al. (2002) stated that:

Textbooks are artifacts. They are a part of schooling that many stakeholders have the chance to examine and understand (or misunderstand). In most classrooms they are the physical tools most intimately connected to teaching and learning. Textbooks are designed to translate the abstractions of curriculum policy into operations that teachers and students can carry out. They are intended as mediators between the intentions of the designers of curriculum policy and the teachers that provide instruction in classrooms. Their precise mediating role may vary according to the specifics of different nations, educational systems and classrooms. Their great importance is constant (p. 2).

Most current mathematics textbooks do not ensure the inclusion of student-cantered mathematical discourse components; rather, they stress on the inclusion of procedural components (Ogan-Bekiroglu, 2007; Stein et al., 2007; Vincent and Stacey, 2008; Skolverket [National Agency of Education], 2011; Boesen et al., 2014). Therefore, in a study of Saudi Arabian mathematics education, it is important to analyze the Saudi Arabian textbooks in light of the inclusion of mathematical discourse components. Moreover, it is also crucial to explore the nature of such an inclusion, if it exists.

Several studies have focused on textbooks analysis in the areas of science and mathematics. For example, Wilson-Lopez and Garlick (2017) conducted a content analysis of students’ writing samples of arguments and discourse. They identified common patterns across students’ writing and used those patterns to propose categories for a rubric that accounted for different dimensions of argumentation specific to engineering. In addition, Jiménez-Aleixandre et al. (2000) found that teachers’ posing of open-ended questions and avoiding traditional teacher-dominated discourse would lead to students’ engagement in higher-quality conversations. Similarly, Mathis et al. (2016) found that teachers’ questioning, by beginning with *why* instead of *what* may develop a more complex thinking in students’ oral discourse. In addition to classroom talk, visual representations are also regarded as an important part of mathematical discourse as they develop students’ academic language skills needed in mathematics learning. This will enable them to make sense of problems through discussion (Shortino-Buck, 2017).

Newell (1990) analyzed the language of mathematical textbooks in his study, discovering many features such as discourse type (narration, description, etc.), coordinators (connectors between sentences), and semantic structures. He stressed that those features may provide a basis for textbook analysis in light of mathematical language. He regarded language analysis, word signifiers, notational signs, and graphical signs as new features to the language features. These are word signifiers, general vocabulary, and word signs used in daily life, as well as mathematical terms, technical vocabulary, special vocabulary, and abbreviations.

Several studies have dealt with the analysis of textbooks regarding the inclusion of some constructs and components that are associated with discourse. Many studies (Pizzini et al., 1991; Chiappetta et al., 2006; Chiappetta and Fillman, 2007; Park et al., 2009; Aldahmash et al., 2016) analyzed school textbooks for the inclusion of different aspects, such as inquiry, which incorporates discourse in its processes, and found that textbooks devoted more text to engaging students in finding out answers, gathering information, and learning how scientists go about their work. Other researchers concluded that science textbooks and workbooks might not be able to help students develop their inquiry components (Wilson-Lopez et al., 2018).

This study explores textbooks’ inclusion of discourse, which aids students’ acquisition of thinking components, habits of persistence, and curiosity. Furthermore, it equips them with self-esteem by instilling confidence in their abilities to succeed in mathematics. Discourse employs scientific dialogue in the development of students’ scientific knowledge (Kuhn, 1970). It may enable students to understand mathematics concepts (Zohar and Nemet, 2001; Chin and Osborne, 2008) and promote their thinking and reasoning abilities (Simon et al., 2008; Gillies and Khan, 2009).

The inclusion of mathematical discourse skills in the textbooks, such as the logical use of words, symbols, diagrams, physical models, and technology, may help students in communicating their ideas and in the development of their meaningful learning, as well as their thinking skill. These procedures may help teachers to structure lessons in such a way to encourage student interaction and assess their students’ mathematical understanding and help students’ present mathematical concepts more precisely, which may develop their thinking skills. Mathematical discourse would also direct students’ conversation during mathematical discussion to ensure the occurrence of meaningful mathematics learning (National Council of Teachers of Mathematics [NCTM], 1991, 2000; 2007; 2014; 2015).

### Purpose of the Study

This study aims to investigate the extent, as well as level, of inclusion of mathematical discourse components in 12th-grade mathematics textbooks and workbooks in Saudi Arabia for the academic year 2019–2020. Through this, the study attempts to find answers to the following questions and subquestions:

(a) Primary Question
  To what extent are mathematical discourse components represented in the 12th-grade mathematics textbooks and workbooks in Saudi Arabia for the academic year 2019–2020?
(b) Subquestions
  What levels of the included mathematical discourse components are represented in the 12th-grade mathematics textbooks and workbooks in Saudi Arabia?
  Which types of activities could contribute most to the promotion of mathematical discourse components in the 12th-grade mathematics textbooks and workbooks in Saudi Arabia?

## Research Methods

Content analysis was employed as a research methodology. In this study, data sources were described, and an analytical framework was then used to explore the representation of mathematical discourse components in the Saudi Arabian 12th-grade mathematics textbooks and workbooks.

### Determining the Levels of Mathematical Discourse

The Mathematical Discourse Analytic Rubric was developed to analyze the targeted mathematics contents. The rubric consisted of five discourse components, as well as the variations of their four levels, depending on the overall number of students or the teacher’s involvement in their learning (Table 1).

**TABLE 1:** Hufford–Ackles mathematical discourse rubric (Hufford-Ackles et al., 2004).

Level	Engagement	Questioning	Explaining mathematical thinking	Mathematical representations	Building student responsibility within the community
1	Content stressing that the teacher dominates the conversation.	Content stressing that the teacher is the only questioner and that questions serve to keep students listening. Content requires the students to give short answers and to respond to the teacher only.	Content questions are focusing on correctness. Students provide short, answer-focused responses. Teacher may give answers as well.	Representations are missing, or the content includes the representations for the students.	Contents encourage students to keep ideas to themselves or to merely provide answers when asked.
2	Content asks the teacher to encourage the sharing of math ideas and directs speakers to talk to the class, not to the teacher only.	Content questions begin to focus on student thinking and less on answers. Only the teacher asks questions.	Content probes student thinking. One or two strategies may be elicited. Content may fill in an explanation and encourages students to provide brief descriptions of their thinking in response to teacher probing.	Content asks students to create math drawings to depict their mathematical thinking.	Content encourages the students to believe that their ideas are accepted by the classroom community. They begin to listen to one another supportively and are now able to restate in their own words what another student has said.
3	Content instructs teachers to facilitate the conversation between students and encourages students to ask questions among one another.	Content asks probing questions and facilitates student-to-student conversation. Students ask each other questions after prompting from the teacher.	Content probes teachers to more deeply learn about student thinking and elicit multiple strategies. Content encourages students to respond to probing, to share their views, and to defend their answers.	Content asks students to label their math drawings so that others are able to follow their mathematical thinking.	Content encourages students to believe that they are math learners and that their, as well as their classmates’, ideas are important. They listen actively so that they can contribute significantly to the discussion.
4	Content encourages students to carry the conversation by themselves. They should only ask teachers to guide students from the periphery of the conversation and to clarify the ideas of others.	Content encourages students to initiate student-to-student conversation. It encourages students to ask questions and to listen to the responses of other students. Many questions begin with “why” and call for justification. It instructs the teacher to ask questions that guide the discourse.	The teacher follows student explanations closely. The teacher asks students to contrast strategies. Students defend and justify their answers with little prompting from the teacher.	Content asks students to follow and help shape the descriptions of others’ mathematical thinking through math drawings. They may suggest edits in others’ math drawings.	Content encourages students to believe that they are math leaders and can help shape the thinking of others. They help shape others’ math thinking in supportive and collegial ways and accept the same support from others.

### Sample: Materials Analyzed

The sample for the content analysis included 12th-grade mathematics textbooks and workbooks (Arabic Edition) adapted from the McGraw-Hill series. These were recently applied in Saudi Arabia in light of the level and the extent of their inclusion of mathematical discourse components. Mathematics documents analyzed in this study included two textbooks and two workbooks for the first and the second terms in the academic year 2019–2020. The mathematics textbooks were 404 pages long (214 for the first term and 190 for the second term) and have eight main chapters altogether. The workbooks were 48 pages long (25 for the first term and 23 for the second term) with eight main sections corresponding to the textbooks’ chapters. Each chapter contained three to seven lessons. There were 42 lessons across the eight chapters. We intentionally selected and analyzed four lessons from each of the textbooks and associated parts of the workbooks—one lesson from each chapter in order to represent a different variety of lessons. Where some lessons included discovery activities such as an introduction to each lesson, others include thinking skills activities or expanded activities as enrichments of the lessons. Many of the activities incorporated a set of problems with the same characteristics. Therefore, we dealt with each of them as one activity. Some activities that included several problems with different characteristics were grouped into sets of activities that have similar characteristics. Many activities have only one problem, and these were dealt with as a single entity. We analyzed the entire selected lesson in each chapter and the related set of activities or problems in the workbook. Each lesson includes an introduction, the concept being studied, examples, and problems.

The main target of the analysis was the conceptual framework used to guide mathematical discourse components. Studies presented a variety of conceptual frameworks for the analysis of printed material from a particular perspective (Chiappetta et al., 2006; Chiappetta and Fillman, 2007; Kahveci, 2009; Dunne et al., 2013; Vesterinen et al., 2013; Aldahmash et al., 2016).

In this study, the mathematics textbooks and associated workbooks were analyzed using the following mathematical discourse components identified by Hufford-Ackles et al. (2004) as this rubric is suitable for this study. Furthermore, we made slight wording changes to the instrument so that it would be suitable for the textbook’s analysis. The rubric includes the following components:

Component 1: engagement
Component 2: questioning
Component 3: mathematical thinking
Component 4: mathematical representations
Component 5: building student responsibility within the community.

There are four levels assigned for each component (ranging from 4, “more student directed,” to 1, “more teacher directed”). The rubric includes five main components and 20 subcomponents, each of which represents a math discourse component and levels to be included in the mathematics curriculum (Hufford–Ackles, 1999).

The rubric was redesigned to fit the analysis of mathematics learning content for the 12th grade (third year of secondary school) in Saudi Arabia. The English version of the rubric was translated and then back-translated to ensure that the evaluators clearly understand the content of the instrument.

## Procedure of the Content Analysis

The following steps were followed in the content analysis of the mathematics learning sources used in this study. First, we identified the analysis categories, which are the discourse components and subcomponents specified in the instruments’ rubric. Thereafter, the mathematics lessons in the textbooks and the related sections in the workbooks were specified as the analysis units. All parts of the lessons were coded by marking the appropriate column cell in the analytical framework. We marked more than one for each analysis unit if necessary. The marks for each component were then counted, organized, and tabulated. Finally, the obtained number was divided by the total number of mathematical discourse components found in each lesson, and the percentages of the frequencies were calculated for each book.

### Reliability of the Content Analysis

In order to ensure the reliability of the data collected for this study, the analyses of the sample content of the 12th-grade mathematics textbooks and workbooks used in Saudi Arabia were assigned to two university math educators. These assigned university math educators served as its raters. The results of their coding of each unit of analysis of the five mathematical discourse components were assessed to ensure that the degree of agreement was reached. The reliability of the analysis value was determined by using the following κ formula developed by Cohen (1990):

k=(po-pc)/(1-pc)

where po represents the proportion of the analysis on which the two raters agree, and pc represents the proportion of ratings for which agreement is reached by chance. We used this formula because it corrects for both the number of categories and the probable frequency with which each is used by the coder; it also considers chance agreement. The percentage agreement between the two raters for activities included in the analyzed secondary school textbooks and workbooks ranged from 73 to 92%, with a corresponding range of κ values from 0.66 to 0.87. According to these values, there is a high degree of agreement between the two raters (Lumpe and Beck, 1996; Chiappetta and Fillman, 2007). Rubinstein and Brown (1984) indicated that κ value range between 0.40 and 0.75 represents fair to good agreement beyond chance.

### Validity of the Analysis

To establish the rating rubric’s content validity as well as fine-tune the rating rubric, a pilot study was conducted ahead of the content analysis. This helped determine the various levels of mathematical discourse components for a small sample of lessons. This step helped us make necessary revisions of the instrument’s rating rubric prior to its implementation, as well as to determine if the rating rubric accurately measured the content (Creswell and Miller, 2000).

We calculated the weighted means as well as the weighted percentages in order to explain the results. These weighted means of responses to the items, which are the measure of central tendency, were calculated based on the number of levels in the rubric (four levels). The range is three, and the length of the category is 3/4, or 0.75. Thereafter, the weighted mean intervals for each level of the rubric, or each level of the inclusion of the mathematical discourse, are as follows: level 1, from 1 to 1.75; level 2, from 1.76 to 2.51; level 3, from 2.52 to 3.08; and level 4, from 3.28 to 4.

## Results

In this part, we presented the data regarding discourse components included in the 12th-grade mathematics textbooks and workbooks and discussed the nature of the results achieved from those data. Table 2 includes frequencies, weighted means, and percentages for the level of inclusion of discourse components in the textbooks and workbooks. The results indicated that components 1 and 4 were included in the analyzed books at the first level, “Content stresses that the teacher dominates conversation.” Components 2 and 3 were included in the books at level 2, “Content questions begin to focus on student thinking and less on answers. Only the teacher asks questions.” Component 5, “Building student responsibility within the community” was not included in the analyzed textbooks and workbooks.

**TABLE 2:** Frequencies, means, and percentages for discourse components included in the 12th-grade mathematics textbooks and workbooks.

Components of discourse	Freq. (f%)				Total	Weighted means	%	Level
	
	1	2	3	4				
Component 1: engagement	45	0	0	0	45	1	25	1
Component 2: questioning	41	190	21	0	252	1.92	48	2
Component 3: explaining mathematical thinking	45	116	92	2	255	2.2	55	2
Component 4: mathematical representations	82	10	29	0	121	1.56	39	1
Component 5: building student responsibility within the community	0	0	0	0	0	0	0	0

Table 3 includes frequency means and percentages for the inclusion of discourse in each of the 12th-grade mathematics textbooks and workbooks. The results indicated that components 1 and 4 were included in the analyzed textbooks at the first level. On the other hand, components 2 and 3 were included at the second level. Regarding the workbook, the results showed that component 1 was included at level 1, whereas components 2, 3, and 4 were included at level 2. Component 5, “Building student responsibility within the community” was not included in the analyzed textbooks and workbooks. The results indicated that the workbooks did not include component 4 (engagement) at any level, as was the case for the textbooks.

**TABLE 3:** Frequencies and percentages of inclusion of each level of discourse components in both students’ textbooks and the workbooks for the 12th-grade mathematics textbooks and workbooks.

Book type	Level	Freq.					Total
		
		Component 1	Component 2	Component 3	Component 4	Component 5	
Textbooks	1	45	41	45	79	0	210
	2	0	159	95	8	0	262
	3	0	19	79	22	0	120
	4	0	0	2	0	0	2
Total		45	219	221	109	o	594
Weighted means		1	1.90	2.17	1.48	0	
Inclusion Level		1	2	2	1	0	
Workbooks	1	0	0	0	3	0	3
	2	0	31	21	2	0	54
	3	0	2	13	7	0	22
	4	0	0	0	0	0	0
Total		0	33	34	12	0	79
Weighted means		1	2.06	2.38	2.33	0	
Inclusion level		1	2	2	2	0	

## Discussion

The results of the analysis indicated that the inclusion of almost all components of the mathematical discourse fluctuated between levels one and two. These results indicate that the inclusion of the discourse component in the textbooks and workbooks is teacher-directed rather than student-directed as specified by the National Council of Teachers of Mathematics [NCTM] (1991). The books neglected component 5, “Building student responsibility within the community.” This component is extremely important for students’ current and future lives, as it builds their ability to be responsible and active members in society. If properly included in the math curriculum, it may encourage students to believe that they are math leaders who can help shape the thinking of others. The proper inclusion may help shape mathematical thinking in supportive, collegial ways and at the same time accept the same support from others (National Council of Teachers of Mathematics [NCTM], 1991; Hufford–Ackles, 1999). Component 3, “Explaining mathematical thinking,” was included in the books at level 2. This type of inclusion deprives students of the ability to respond to probing questions, share their views, and defend their answers, with little prompting from the teacher (National Council of Teachers of Mathematics [NCTM], 1995, 2000). Some mathematical problems were assigned at level three in component 3, because the content asked students to justify their answers. For instance, in the math textbook for term 1, problem 25, (p. 15). However, the ultimate inclusion was found at level 2. Component 2 “questioning” was included in the first level. It should be included at the fourth level to encourage students to initiate talk among themselves and encourage them to ask questions and listen to responses. Many questions ask “why” and call for justification. The content should instruct teacher to ask questions that guide students’ discourse (Chin and Osborne, 2008). The content did not encourage any type of student-to-student questioning and conversation. Sometimes there was a high level and depth to questions, such as question or problem number 39, page 16, of the textbook for term 1. However, these questions could not be categorized as high level because they did not encourage students to talk with each other or with the teachers. This might result in students being close-minded and not socially active. Engagement is crucial to student success. If engagement was included in the math book so that students were encouraged to carry the conversation themselves, they might be able to implement tasks to facilitate meaningful mathematical discourse themselves (Hufford-Ackles et al., 2004; National Council of Teachers of Mathematics [NCTM], 2014, 2015). Proper inclusion of engagement may enable students to be attached to the concept being learned and hence will facilitate better understanding.

Regarding the inclusion of component 3, “Explaining mathematical thinking,” the inclusion did not exceed that of level 2, because none of the activities probed teachers to learn more deeply about students’ thinking or to elicit multiple strategies. The content did not encourage students to respond to probing, share their views, or to defend their answers. For example, high-thinking problems in the textbook for term 1 (p. 179) asked students to interpret their justification but did not ask them to express their opinion to their peers. Most of the mathematical representations were included at level 1. None of these asked students to label their math drawings so that others are able to follow their mathematical thinking, or to follow and help shape the descriptions of others’ math thinking through math drawings and suggest edits to others’ math drawings (National Council of Teachers of Mathematics [NCTM], 1991, 2000; Hufford–Ackles, 1999). In addition, student’s textbooks did not provide representations for students or ask them to create them; nor did they refer students to a source for such drawings. For example, in textbook 6, term 1 (p. 95), question 11, it was expected that the mathematical model or a drawing of the cell should be included to help students visualize the concept. It is noticeable that the workbooks did not include component 1 (engagement), because the problems were included without any introduction or referral to the textbooks. In view of problems or activities, for instance, in exercise 52 on page 21 of the textbook for term 2, the representations were categorized as level 3 because the content asked students to label these representations. Some lessons included discovery activities, whereas others included thinking skills activities. However, none of these activities provided the opportunity for students to take part in mathematical knowledge activities; for instance, textbook for term 1, page 157. However, the inclusion of these components in the mathematics textbooks and workbooks was found at a low level. This kind of inclusion would not meet NCTM standards and hence would not help students achieve a meaningful understanding of mathematical concepts.

## Conclusion and Implications

The results showed that both Arabic mathematics textbooks and workbooks used in Saudi Arabia did not include appropriate discourse components or skills. Both failed to exceed the second level of inclusion, which will not help learners meaningfully understand mathematical concepts or become active, successful leaders of their community. None of the activities in the 12th-grade mathematics textbooks and workbooks in Saudi Arabia contributed to the promotion of mathematical discourse components. This implies that mathematics textbooks and workbooks should be revised to include mathematical discourse so that this inclusion is more student directed than teacher directed. The inclusion of mathematical discourse skills in the mathematics textbooks would help facilitate mathematical learning among students. This implies also that teachers should be trained to use mathematical discourse in their teaching and strive to develop this discourse among students, even if textbooks and workbooks do not include these skills. The instrument used in this study must be used to conduct studies examining the inclusion of mathematical discourse in mathematics curricula for elementary and middle schools, to widen the literature.

## Data Availability Statement

The datasets generated for this study are available on request to the corresponding author.

## Author Contributions

Both authors listed have made a substantial, direct and intellectual contribution to the work, and approved it for publication.

## Conflict of Interest

The authors declare that the research was conducted in the absence of any commercial or financial relationships that could be construed as a potential conflict of interest.
